# Extracellular vesicles: potential applications in cancer diagnosis, prognosis, and epidemiology

**DOI:** 10.1186/s12907-015-0005-5

**Published:** 2015-04-15

**Authors:** Mukesh Verma, Tram Kim Lam, Elizabeth Hebert, Rao L Divi

**Affiliations:** grid.48336.3a0000000419368075Epidemiology and Genomics Research Program, Division of Cancer Control and Population Sciences, National Cancer Institute, National Institutes of Health, Rockville, MD 20850 USA

**Keywords:** Cancer, Diagnosis, Epidemiology, Extracellular vesicles, Exosomes, Microvesicles

## Abstract

Both normal and diseased cells continuously shed extracellular vesicles (EVs) into extracellular space, and the EVs carry molecular signatures and effectors of both health and disease. EVs reflect dynamic changes that are occurring in cells and tissue microenvironment in health and at a different stage of a disease. EVs are capable of altering the function of the recipient cells. Trafficking and reciprocal exchange of molecular information by EVs among different organs and cell types have been shown to contribute to horizontal cellular transformation, cellular reprogramming, functional alterations, and metastasis. EV contents may include tumor suppressors, phosphoproteins, proteases, growth factors, bioactive lipids, mutant oncoproteins, oncogenic transcripts, microRNAs, and DNA sequences. Therefore, the EVs present in biofluids offer unprecedented, remote, and non-invasive access to crucial molecular information about the health status of cells, including their driver mutations, classifiers, molecular subtypes, therapeutic targets, and biomarkers of drug resistance. In addition, EVs may offer a non-invasive means to assess cancer initiation, progression, risk, survival, and treatment outcomes. The goal of this review is to highlight the current status of information on the role of EVs in cancer, and to explore the utility of EVs for cancer diagnosis, prognosis, and epidemiology.

## Introduction

### Conventional biomarkers and circulating biomarkers

Living cells secrete a large number of endocytic or plasma membrane vesicle including exosomes, microvesicles (MVs), and apoptotic bodies into extracellular space [1-3] called extracellular vesicles (EVs). Different names are reported in the literature for EVs, such as exosomes, ectosomes, oncosomes, apoptotic bodies, microparticles, and microvesicles (MVs) [3]. Based on intracellular origin or biogenesis, two major classes of EVs are reported: exosomes and MVs [4].

The exosomes, nano-particle size (30–100 nm) vesicles with a buoyant density of 1.13 – 1.19 g/cm^3^, are shed by both healthy and diseased cells. Exosomes are derived from the endolysosomal pathway and originate from the endosomal compartment called multivesicular bodies. Lee et al. reported that EVs may be generated by mesenchymal stem cells and their involvement in suppressing angiogenesis [5]. Other cell types, such as platelets, neutrophils, reticulocytes, macrophages, megakaryocytes, monocytes, B and T cells, mast cells, and endothelial cells, release EVs [6].

The microvesticles are generated by budding from the plasma membrane. The microvesicles are 100–1000 nm in size and originate from budding and fusion of plasma membrane into extracellular space, and share several similarities with the parental cells, including having membrane lipids, receptors, and several types of nucleic acids and proteins [1,7-10].

In general, the molecular composition of each EV closely mimics the parental cell or tissue and contains growth factors, receptors, proteases, adhesion molecules, microRNAs (miRNAs), proteins, and lipids [1,7,8]. Several studies reported the presence of numerous bioactive proteins, nucleic acids, lipids and other biomolecules in the EVs [1,11,12]. Caby et al. demonstrated that EVs contain tetraspanin molecules such as CD9, CD63, and CD81; class I and class II major histocompatibility complex (MHC) molecules; and lysosomal-associated membrane protein-2 (LAMP-2) [7]. Thakur et al. demonstrated the presence of double stranded DNA (representing whole genome) inside exosomes isolated from myeloid leukemia, colorectal carcinoma, and melanoma cells [13].

Cancer development is a multistep and multifactorial process that includes uncontrolled growth, resistance to apoptosis, genetic and epigenetic changes, and alterations in the surrounding microenvironment. These changes include the development of EVs and some have speculated that EVs can be potentially used as biomarkers for disease diagnosis, prognosis, and epidemiology [3,4,14-19]. Generally biomarkers indicate the turbulence in the normal biological status that contributes to carcinogenesis. Currently, a number of biomarkers, mostly from circulating cells, are used in diagnosing cancer [15-19], epidemiology [20-23], and treatment follow up [24-26]. Many of the existing biomarkers offer insufficient information about the tissue origin and thus it is difficult to use them in targeted therapy [16]. In turn, this significantly limits their utility in both research and the clinical setting.

Inherent characteristics of EVs may make them ideal next-generation biomarkers for the 21st century research and therapy. The involvement of EVs in intracellular communication and the dynamic nature of their composition, for example, have allowed investigators to explore their tumor-modulating potential [1,4,27-29]. It has been shown that the quantity and composition of EVs changes during cancer development [1,3,30]. For example, Baran et al. reported elevation of number of EVs in gastric cancer patients and the number correlated with the stage of cancer development [31]. Increased expression of CCR6 and Her-2/neu was also observed in plasma samples from patients with advanced stage of the disease. Levels of PTEN present inside exosomes isolated from prostate cancer patients correlated with the development of disease and used for prostate cancer diagnosis [32]. Furthermore, EVs have excellent biodistribution and biocompatibility [33]; these qualities make them ideal for use in drug delivery and distribution. The fact that EVs contain functional nucleic acids suggests that they are like viral particles [34].

EVs have been found in several biofluids including amniotic fluid [35], breast milk [36], bronchoalveolar lavage [37], cerebro-spinal fluid [38], malignant ascites [39], plasma [40], saliva [41] and urine [42]. The ubiquity of EVs in the biofluids, coupled with the fact that they reflect the composition of their parent cells, offer a unique platform for population-based research. It is biologically plausible that EVs could contribute significantly and prospectively to the characterization of normal versus disease states. For example, in epidemiologic research it is challenging and in some cases not feasible to collect multiple biospecimens prior to the development of a disease in large prospective cohort studies. With EVs, epidemiologists could obtain a serial collection of biofluids containing EVs via a non-invasive or minimally invasive approach from both controls and cases. Morphological, molecular and functional analysis of these EV-riched biofluids could be conducted and expand our understanding of cancer risk and development. Investigators have shown differential molecular profiles of EVs in cancer patients’ sera/plasma from breast [43-46], prostate [32,47-50], lung [51], liver [52], gastric [53], glioblastoma [54,55], KSHV-associated malignancies [56]; and urine from prostate [57-59].

Table 1 presents a list of tumor types in which EVs have been reported, and Table 2 provides a list of biospecimens in which EVs may be isolated for diagnosis, prognosis, and epidemiology. Viaud et al. and other groups proposed using EVs as a cell-free vaccine in therapeutics [5,60].

**Table 1:** **Extracellular vesicles’ analysis in different tumor types**

**Cancer types**	**Comments**	**References**
Bladder cancer	Exosomes from urine contain the angiogenesis-promoting protein EDIL-3	[99]
Breast cancer	Microvesicle numbers and the amount of focal adhesion kinase and EGFR in plasma fractions were associated with different stages of breast cancer	[89]
Colorectal cancer	Proteomic analysis was conducted on EVs from colorectal cancer cells	[103]
Gastric cancer	Proteomic analysis was conducted on EVs from gastric cancer patients	[31]
Glioblastoma	Microvesicle RNA biomarkers of glioblastoma multiforme were identified	[29,55]
Head and neck cancer	Exosomes and microvesicles from patient saliva were used for diagnosis	[104]
Lung cancer	Proteins isolated from microvesicles in pleural effusions due to lung cancer were characterized to identify diagnostic markers	[3]
Melanoma	Proteomic analysis was conducted on exosomes from melanoma patients were used for	[105]
Ovarian cancer	Exosomes from ovarian cancer patients contain different sets of proteins and miRNAs compared to exosomes from normal subjects; the amount of circulating exosomes was 4 times higher in patients	[1,2,92,93,106]
Pancreatic cancer	EVs were used in diagnosing pancreatic cancer	[30]
Prostate cancer	Urine exosomes expressed higher levels of PCA-3 and TMPRSS2-ERG	[58]

**Table 2:** **Biofluids/biospecimens used to isolate extracellular vesicles for cancer diagnosis, prognosis, and epidemiology**

**Biospecimen**	**Comments**	**References**
Ascites	Used in diagnosing ovarian cancer and determining its aggressiveness	[39]
Blood and plasma	Used in diagnosing ovarian cancer and breast cancer	[89,93]
Breast milk	Exosomes were isolated from breast milk	[36]
Mesenchymal stem cells	Suppression of angiogenesis shown in tumor cells mediated by miR-16 isolated from exosomes	[5]
Pregnancy-associated sera	Exosomes were isolated in different stages of pregnancy	[107]
Saliva	Exosomes and MVs found in patient saliva	[104]
Stem cells	Renal stem cells contained MVs with angiogenesis-specific mRNAs and miRNAs	[94]
Tissues	Ovarian cancer tissues were used to isolate exosomes and then in isolating miRNAs from them	[1,14]
Urine	Urine was used to isolate exosomes and in analyzing proteins by MS or transcriptome analysis	[58,99]

## Review

### EV biogeneration

There are two main pathways of exosomal generation, endocytic and exocytic. According to the endocytic pathway, exosomes are formed in a two step process and are released from the plasma membrane via the endosomal sorting complex required for transport (ESCRT) [61]. The second pathway is ESCRT independent and require sphingolipid (ceramide) [62], tetraspanins [63], and heat shock proteins [64]. Ghossoub et al. characterized proteins such as GTPase ADP ribosylation factor 6 (ARF6) and its effector phospholipase D2 (PLD2) involved in biogenesis of exosomes [65]. These two proteins control budding of intraluminal vesicles into multivesicular bodies. Few other proteins involved in biogenesis process include TSG101 [66,67], Rab5 [68], Rab7 [69], Vps4, and Vps36 [70]. Among them TSG101 is an integral part of the ESCRT (this complex includes ESCRT-0, ESCRT-I, ESCRT-II, ESCRT-III, ALIX, and TSG101 [71]). ALIX promotes intraluminal budding of vesicles in endosomes upon interaction with syntenin which is the cytoplasmic adapter of heparin sulfate proteoglycan receptor [72].

Different types of EVs are generated from endosomes and during maturation of the endosomes they accumulate intraluminal vesicles. Protein sorting in these vesicles depends on monoubiquitination and the endosomal sorting complex required for transport. mRNA recruitment is guided by a zip code in the 3'UTR, and miRNA by physical and functional coupling of RNA-induced silencing complexes (RISCs). MV as opposed to exosomes synthesis involves an increase in cytosolic Ca++, which activates different pathways, resulting in depolymerization of the actin cytoskeleton and, finally, the release of MVs [6]. In HPV-positive cells, silencing of the E6/E7 proteins resulted in alterations in the number and composition of MVs [73]. MVs from HPV-positive cells contained higher levels of survivin and anti-apoptotic proteins compared to HPV-negative cells. In lung cancer, MVs are released in circulation and in pleural effusion due to lung cancer [3]. About 900 MV specific proteins were identified from pleural effusion of nonsmall cell lung cancer patients. These proteins were different from EV proteins isolated from patients with other cancer types. Park et al. developed bioinformatics tools and identified pathologically-significant proteins from those 912 MV specific proteins from lung cancer patients [3]. Ostrowski et al. demonstrated the role of Rab27a and Rab27 b in exosome secretion pathway [74].

### EV functions

Various biological roles have been proposed for EVs, including disposal of superfluous or harmful cellular content, emission of signaling and regulatory molecules for cell-cell communication and functional modification, propagation of pathogens, stimulation or inhibition of the immune system, antigen presentation, and many more [4,75]. EVs are involved in both beneficial and pathological functions. EVs can be used in cancer diagnosis and prognosis precisely because the function of the original cell can be extrapolated by examining EV composition (proteins, mRNAs, miRNAs, non-coding RNAs, lipids, and other molecules) in body fluids. Kahlert et al. demonstrated the presence of double stranded DNA in serum collected from pancreatic cancer patients [9]. DNA isolated from these EVs was used to detect p53 and KRAS mutations. Collectively, EVs were not shown to contain mitochondrial, nuclear, or endoplasmic reticulum proteins [76,77]. Exosomes, specifically, do not contain most of the ribosomal RNAs and contain mainly mRNAs and miRNAs [58]. EVs can interact with target cells directly via cell surface receptors, or can be internalized by target cells via membrane fusion or endocytosis [78]. Once EV-associated signaling molecules are recognized, the target cell’s function is modified or regulated.

### EV isolation and analysis of the contents

Table 3 shows the methods that are used for isolating extracellular vesicles. Obtaining pure exosomes is critical for preserving the physicochemical and functional characteristics of the exosomes. Most frequently used and accepted method for enriching EVs is ultracentrifugation [79] followed by density gradient centrifugation [80], however, these procedures are laborious and time consuming (2–24 h). Moreover, protein aggregates may co-precipitate with exosomes and there is a possibility of labile biomolecules being degraded during the long centrifugation steps. Another limitation is that these methods do not offer size separation. Alternatively, size based filtration is another approach for fractionating and obtaining exosome-rich preparations [81,82]. These filter- based methods are quick (<2 h) at isolating exosomes; nevertheless, they have been shown to isolate nano-particle size RNA-protein complexes and chylomicrons whose size (100–350 nm) is similar to the exosomes as well, thus raising concerns about contamination. Currently, polyethylene glycol or other polymers based precipitation is being extensively commercialized as an alternative to isolating exosomes. These precipitation methods are quick (~2 h), but non-specific because they are known to also isolate non-exosome biomolecules and cellular debris. Additionally, affinity methods based on capture of exosomes using peptides or antibodies are being used as specific methods.

**Table 3:** **Methods for isolating extracellular vesicles (EVs)**

**EV Isolation Method**	**Sample**	**~ Time required for isolation**	**Reference/Company**
Deferential centrifugation	Serum, urine and cell culture supernatant	2-15 h	[79]
Deferential centrifugation	Cell culture supernatant or saliva	2-15 h	[108,109]
Density gradient centrifugation	Cell culture supernatant	24 h	[80]
Sequential membrane filtration	Cell culture supernatant and biofluids	24 h	[81,110]
Nanomembrane	Urine	<2 h	[82]
Size exclusion chromatography	Plasma	20 min	[111]
Microfluidics	Cell culture supernatant	-	[112]
Nanoshearing	Cell culture supernatant and serum	-	[46]
ExoCap	Cell culture supernatant and biofluids	30 min	MBL International
ExomiR	Cell-free biofluids’ exosomal RNA	20 min	Bioo Scientific
Exo-spin	Cell culture supernatant, urine and saliva	3 h	Cell Guidance Systems
ExoQuick	Serum, plasma, ascites, urine, CSF and cell culture supernatant	2-15 h	System Bioscinces
miRCURY	Serum, plasma, cells, urine and CSF	2 h	Exiqon
Total exosome isolation	Cell culture supernatant and biofluids	2 h	Life Technologies
PureExo	Serum, plasma and cell culture supernatant	2 h	101Bio
ME Kit based on Vn96 peptide binding to heat shock proteins on exosomes	Serum, plasma, urine, cell culture supernatant	30 min	New England Peptide
Streptavidin-biotin-specific antibody to a known antigen on exosome	Cell culture supernatant	>12 h	Life Technologies
Anti-tetraspanin antibody-magnetic bead based	Cell culture supernatant	>12 h	Life Technologies
Anti-EpCAM-antibody magnetic bead based	Cell culture supernatant	>4 h	Life Technologies

The current technologies are thus limited, and moving forward, simpler methods are needed. Improved methods must be able to combine isolation of pure exosomes and analysis, since the existing methods are not perfect and yield varying compositions and quality of exosomes. Microfluidics combined with biosensors, employing biological or biomimetic capture agents, may quickly isolate total or specific exosomes and facilitate faster and cheaper analysis of exosomal cargo. This technology may advance our understanding of EVs, and increase EVs utility in research and therapy.

Methods to characterize EVs are emerging and developing. Extracellular vesicles have been routinely characterized using transmission electron microscopy (TEM), florescence microscopy or flow cytometry [83]. There are several limitations to TEM. It is expensive and requires specialized experience, the sensitivity of the fluorescence microscopy is not optimal for nano-micro vesicle characterization. Nanoparticle tracking analysis (NTA), designed to measure the size and concentration of EVs, is based on flow cytometry. NTA has been reported to be used for analyzing circulating EVs [84]. Wei et al. established a method called electric field induced release and measurement (EFIRM) for simultaneously disrupting and releasing exosomal RNA/biomarkers for on-site monitoring [85]. They have adapted the method for blood and saliva [85]. A multiplexed microfluidic device for specific capture and detection of exosomal contents using nanoshearing technology, based on a tunable alternating current electrohydrodynamic (ac-EHD) methodology, was used for measuring human epidermal growth factor receptor 2 (HER2) and PSA in human samples [46]. Ueda et al. developed anti-CD9 antibody-coupled to highly porous monolithic silica microtips for exosome extraction from multiple clinical samples, and applied it to measure exosomal proteins by mass spectrometry in serum samples from lung cancer patients [51].

Recent estimates indicate that 1 ml serum (or comparable amounts of other biofluids) is sufficient to analyze EV miRNAs and proteins [30]. Park et al. isolated 912 proteins from MVs from the pleural effusion of non-small lung cancer patients by applying nuclear magnetic resonance mass spectrometry (NMR-MS) followed by SDS-PAGE [3]. The composition of MVs from these lung cancer patients was different than MVs from other malignancies such as breast, prostate, and colon cancers. Further characterization of these proteins (mainly lung-enriched surface antigens and proteins related to epidermal growth factor receptor [EGFR] signaling) indicated their potential in lung cancer diagnosis. Ovarian cancer ascites were isolated by centrifuging malignant ascites to remove cells, followed by sucrose gradient centrifugation [39]. The pelleted vesicles were further characterized by gelatin zymography [86]. Ascite-derived exosomes exhibit gelatinolytic activity, and gelatinases increase tumor progression [87]. Analysis of EV proteins was achieved by liquid chromatography combined with matrix-assisted laser ionization/desorption time of flight mass spectrometry (LC-MALDI-TOF MS) [88]. Specific MV proteins, such as focal adhesion kinase and EGFR, from breast cancer patient sera were characterized by Western blotting [89].

### Pathological functions of exosomes and utility in cancer research

In response to pathological alterations, cells communicate with each other by secreting a heterogeneous mixture of vesicles (including MVs and exosomes) with different compositions. Pegtel et al. demonstrated that miRNAs present in the exosomes isolated from Epstein-Barr Virus (EBV)-infected cells could release their miRNA (and induce silencing of specific genes) when kept in contact with surrounding uninfected B cells [90]. Whether EVs are released from the infected host or infectious agent, information from the analysis of EVs may facilitate their use as biomarkers for diagnosis. The EV content analysis also will promote understanding of host-infectious agent interaction to enable effective vaccine design and development of novel therapeutics [91].

### Using EVs to diagnose cancer

EVs’ contents reflect the content of the cells (both stromal and tumor) from which they originate. EVs could be used as biomarkers for diagnosis or to predict or monitor a patient’s response to treatment. It has been demonstrated, at least in the case of ovarian cancer, that the profiling of miRNAs isolated from circulating exosomes is similar to exosomes present in tissue [14]. This suggests that circulating exosomes can be used as a surrogate for tissue miRNAs, and exosome-derived miRNA profiling can be used as a diagnostic biomarker for ovarian cancer.

Differential prevalence of EVs has been identified in varying tumor types (Table 1) which could be exploited for future research. For example, diagnostic biomarkers for ovarian cancer are currently unknown and approximately 70% of ovarian cancer cases are diagnosed at an advanced stage. Emerging evidence suggests that exosome biomarkers may be useful in ovarian cancer screening [2]. Specifically, Went et al. showed that the amount of circulating exosomes released from tissues is four times higher in ovarian cancer patients than in normal subjects [92]. Liang et al. characterized the proteomic and genomic profiling of ovarian cancer exosomes [2]. This study showed that epithelial cell adhesion molecule (EpCAM), CD24, and miRNAs are present in the exosomes. These contents may serve as additional biomarkers for use in diagnosing ovarian cancer. Since EpCAM overexpression was reported to be correlated with epithelial cell proliferation, a technology using microbeads coated with EpCAM antibodies may be used for isolating ovarian cancer exosomes for further analysis [93]. Taylor et al. demonstrated that exosomal miRNA profiling can be used as diagnostic biomarkers for ovarian cancer [14]. This study showed that a group of miRNAs (miR-21, miR-141, miR-200a, miR-200b, miR-200c, miR-205, and miR-214) present in the exosomes possessed characteristics similar to those isolated from ovarian tissue. Furthermore, higher levels of these miRNAs are correlated with advanced ovarian cancer [14].

In addition to exosomes, the diagnostic promise also holds for microvesicles in several malignancies. Elevated levels of MVs were reported in sera from breast cancer patients compared to normal individuals [89]. In renal cancer, MVs containing different miRNAs and mRNAs were released from renal cancer stem cells, and their role in tumor vascularization has been proposed [94,95]. To identify an EV biomarker for glioblastoma multiforme, serum MVs were analyzed by microarray expression analysis, and a group of RNAs that are upregulated or downregulated in this tumor type was identified [29,55]. Transcriptome analysis of urine exosomes from prostate cancer patients showed higher levels of PCA-3 and TMPRSS2-ERG when compared with controls [58]. Because prostate cancer tissue is heterogeneous in its phenotypes, a biopsy taken from a specific site may not represent the overall tumor malignancy status, including tumor-specific variants, mutations, and levels of mRNAs and miRNAs for diagnosing prostate cancer. Transcriptome analysis of circulating vesicles, however, may be representative of prostate cancer malignancy status.

### Using EVs to determine cancer aggressiveness

Identification of cancer aggressiveness is an important marker in prognosis. There is promising evidence that EVs can be capitalized for this purpose [96]. For example, in ovarian cancer, higher levels of CD24 indicate worst prognosis and reduce patient survival rates [97]. A study showed that levels of EpCAM and CD24 present in exosomes were correlated with the aggressiveness of ovarian cancer [39]. In that study, Runz and colleagues isolated ascites-derived exosomes from ovarian cancer patients. They found that cytoplasmic localization of CD24 occurred in tumors with high invasive potential. In melanoma, exosomes isolated from metastatic cells were capable of making primary tumor aggressive by permanently converting bone marrow progenitors [96]. Proteins involved in regulation of membrane trafficking and exosome formation, such as RAB1A, RAB5B, RAB7, and RAB27A, were highly expressed in these melanoma cells. In bladder cancer, exosomes isolated from the urine of high-grade bladder cancer patients had higher levels of EDIL-3, a molecule that promotes angiogenesis, compared to exosomes from healthy individuals [98,99]. The line of evidence extends to hepatocellular carcinoma where EDIL-3 was shown to be overexpressed in tumors [100]. EDIL-3 plays a role in tumor progression. Receptor tyrosine kinase MET mediates exosome mediated metastatic behavior in the melanoma mice model. When Met expression was reduced in exosomes, the pro-metastatic behavior of bone marrow cell also diminished [96].

### Using EVS for therapeutic purposes

As mentioned earlier, applying EVs in therapeutics as a cell-free vaccine was proposed by Viaud et al. and others [5,60]. Advantages of EVs include that they are non-living and easily recovered from biological fluids. These are bioavailable vehicles that are well tolerated; targetable to specific tissues; resistant to metabolic processes; and, most-importantly, membrane-permeable. EVs are considered ideal candidates for delivery of miRNAs/small interfering RNAs (siRNAs), or drugs that otherwise would be degraded rapidly. Potential applications of EVs in cancer therapeutics also were proposed recently [4]. EVs can be loaded with a selective combination of drugs for delivery with minimal issues related to immunity and passing the blood–brain barrier [101]. EVs are stable in the blood and can deliver functional RNAs to host cells. For personalized medicine, EVs collected from one individual can be enriched, mixed with a drug(s), and given back to the same individual without causing immunity-related issues. EVs from tumors carry tumor antigens and present them to T cells, priming them to induce the anti-tumor response and resulting in tumor cell death [27,28]. Using EVs, especially nanovesicles, in cell-free cancer vaccines also has been proposed [101]. EVs are capable of priming the immune system to recognize tumor-specific antigens and initiate an appropriate immunologic response toward the abnormal cancer cell while leaving the normal surrounding cells unchanged.

### Potential utility of EVs for population-based research

Beyond clinical and therapeutic usefulness, EVs can be exploited for population-based research in several ways. For example, the bioavailability of EVs (from milk, urine, blood, serum, etc.) may be capitalized for epidemiologic research in which longitudinal studies are difficult to conduct using tissue biospecimens. EVs offer a non-invasive and almost continuous access to circulating information on the disease state in epidemiologic investigations. In addition, cancer epidemiologists may use EVs to investigate whether the characteristics of EVs may be influenced by exposure to certain carcinogenic factors (e.g., smoking, physical activity, obesity). In turn, EVs may be used as biomarkers in epidemiologic studies to characterize the mechanistic underpinnings and follow up on findings from association studies. There is evidence to support EVs utility. Data from a study by Xu et al. [102] showed that arsenic-transformed human bronchial epithelial cells release exosomal miR-21 that subsequently stimulated normal neighboring cells. These data suggest that neoplastic cell-to-normal cell communication mediated by an exosomal miRNA may be involved in carcinogenesis induced by exogenous factors.

### Challenges and potential solutions

Although the concentration of EVs increases in cancer, the methods for EV isolation tend to be time-consuming and yield samples that need further purification. These factors, together with the high costs currently associated with this process, may limit research—particularly in epidemiologic studies in which thousands of samples are analyzed. Therefore, further improvements in EV isolation, purification, and content analysis are required. Further, current isolation technologies make it difficult to distinguish different EV subpopulations. This explains the broad use of the term “EVs” in publications instead of specific types of EVs (e.g., exosomes and MVs). Contamination from RNA-protein complexes, protein aggregates, and other micro particles may affect the results. Therefore, there is a need for technologies which can isolate highly pure EVs for downstream analysis (transcriptomics, miRnomics, proteomics).

Because of the multifunctional nature of EVs, it is important to understand the balance between healthy and oncogenic EV signaling. One way to use EVs for therapeutic purposes is to remove these vesicles to prevent metastasis and tumorigenesis. However, the technical and financial challenges involved in removing EVs have prevented the clinical implementation of this technique to date. Technological improvements being made may change this in near future.

At times, MVs released from a specific organ site exhibit the properties of drug resistance by carrying multidrug resistance (MDR) proteins [6]. There is a need to understand how EV biosynthesis and/or intercellular communication can be altered so that EVs can be used for targeted therapies. Research also is needed to evaluate whether therapies that target the uptake of tumor-derived EVs by recipient cells are specific enough to prevent side effects. A better understanding of the molecular mechanisms that underlie EV biosynthesis and their physiological relevance also is needed.

## Conclusions

Despite the challenges cited above, the scientific community is interested in these tissue-derived vesicles and their multiple roles and functions. EVs hold great promise for cancer diagnosis and treatment. Likewise, there are many potential uses of EVs in cancer research and epidemiology.

## Abbreviations

EGFR: Epidermal growth factor receptor
EpCAM: Epithelial cell adhesion molecule
ESCRT: Endosomal sorting complex required for transport
EVs: Evesicles
mRNA: messenger RNA
miRNA: microRNA
MVs: Microvesicles
